# Predicting role of Myc-induced nuclear antigen 53 in determining the development and severity of systemic lupus erythematosus

**DOI:** 10.3389/fimmu.2024.1370738

**Published:** 2024-05-28

**Authors:** Batool Zamani, Ehsan Dadgostar, Hossein Akbari, Hossein Motedayyen, Hassan Nikoueinejad

**Affiliations:** ^1^ Autoimmune Diseases Research Center, Kashan University of Medical Sciences, Kashan, Iran; ^2^ Students’ Research Center, Kashan University of Medical Sciences, Kashan, Iran; ^3^ Trauma Research Center, Kashan University of Medical Sciences, Kashan, Iran; ^4^ Nephrology and Urology Research Center, Baqiyatallah University of Medical Sciences, Tehran, Iran

**Keywords:** systemic lupus erythematosus (SLE), disease severity, Myc-induced nuclear antigen (Mina) 53, Th17 cells, regulatory T cells (Tregs)

## Abstract

**Introduction:**

Systemic lupus erythematosus (SLE) as an autoimmune disease can relate to an imbalance between regulatory T cells (Tregs) and Th17 cells. Previous reports have shown that Myc-induced nuclear antigen (Mina) 53 protein is involved in the developments of Tregs and Th17 cells. Therefore, the current study focused on determining whether Mina53 level is correlated to the severity of SLE.

**Methods:**

The blood samples were collected from 60 patients with SLE (30 cases with mild SLE and 30 cases with severe SLE) and 30 healthy subjects. The serum concentration of Mina53 was measured using enzyme-linked immunosorbent assay (ELISA). The expression of Mina53 gene was assessed using real-time PCR method after extracting RNA from isolated peripheral blood mononuclear cells and synthesizing cDNA.

**Results:**

Patients with SLE showed significant increases in the serum level and gene expression of Mina53 compared to healthy subjects (P<0.001). Furthermore, serum level and gene expression of Mina53 showed significant effects on SLE disease and its severity (P<0.01). There was the highest sensitivity and maximum specificity in the cut-off point of Mina53 serum level equal to 125.4 (area under the curve (AUC)=0.951) and Mina53 expression level equal to 8.5 (AUC=0.88) for SLE diagnosis. The cut-off point of Mina53 serum level equal to 139.5 (AUC=0.854) and the cut-off point of Mina53 expression level equal to 8.5 (AUC=0.788) had the highest sensitivity and maximum specificity determining severe forms of SLE.

**Discussion:**

Our results showed that the changes in serum and expression levels of Mina53 have significant effects on SLE disease and its severity. These levels may be considered as diagnostic and predictive markers for SLE.

## Introduction

Systemic lupus erythematosus (SLE) is a chronic autoimmune disease caused by a variety of hormonal, environmental, and genetic factors (1–3). Its global prevalence is estimated to be 43.7 (15.87 to 108.92) per 100,000 individuals. The value in women was 78.73 (28.61 to 196.33) per 100,000 people, while the estimate in men was 9.26 (3.36 to 22.97) per 100,000 persons (4, 5). It is characterized by the presence of different autoantibodies, mainly anti-nuclear antibodies (ANAs), which may participate in its wide range of clinical symptoms and complications (6) such as nephritis (7), optic neuromyelitis (8), pericarditis (9), atherosclerosis (10), and cardiovascular complications in which the latter leads to death (11).

Myc-induced nuclear antigen (Mina) 53 gene, known as mineral dust-induced gene (Mdig) and ribosomal oxygenase 2 (RIOX2), is a Jumonji C (JmjC) domain-containing 2-oxoglutarate (2OG)-dependent oxygenase localizing to the nucleolus. It modifies ribosomal proteins through hydroxylation of amino acids (12). Mina53 is transcriptionally stimulated by the c-myc oncoprotein, which is mostly known as a proto-oncogene playing some regulatory roles in the cell growth of several solid and hematological cancers (13–21). These physio-pathologic roles are not restricted to cancers. There are few studies pointing to the increased expression of Mina53 in some allergic and autoimmune reactions (22, 23). Mina53, as a transcriptional co-repressor of the interleukin-4 (IL-4) encoding gene (23), shifts immune responses toward T helper 2 (Th2) cells, which play fundamental roles in atopic pulmonary inflammation and parasitic worm expulsion (24). The role of Mina53 in the pathogenesis of autoimmunity may be due to its positive effects on inflammatory responses of Th17 cells (25).

Immune imbalance has a pivotal role in the development of diseases with immune pathophysiology (26, 27). The imbalance between Th17 cells and regulatory T cells (Tregs) may contribute to the pathogenesis and development of SLE. Patients with SLE have an increased number of Th17 cells and enhanced production of IL-17, which are related to disease severity (28, 29). It is shown that Mina53 induces IL-17 expression and reduces Foxp3 expression as the main transcription factor for the development and function of Tregs (30). Having considered that Mina53 affects the expression of gene(s) involved in the pathogenesis of SLE and thereby participates in various autoimmunity and allergic reactions, the current study investigated whether changes in the serum and expression values of Mina53 may serve as biomarkers in predicting SLE development and determining its severity.

## Materials and methods

### Study populations

The study population consisted of 60 patients with SLE (30 cases with mild and 30 subjects with severe forms of the disease) and 30 healthy subjects. The diagnosis of SLE was confirmed by an internal medicine specialist using systemic lupus international collaborating clinics classification criteria (31). Patients were interviewed by the specialist, and the disease activity index (DAI) was collected using a questionnaire according to the SLEDAI-2K (30-day) guideline, as previously described (1). According to the questionnaire, scores between 6–12 and more than 12 were considered mild and severe forms of the disease, respectively.

Inclusion criteria were the absence of health problems, malignancy, and other disorders affecting the immune system in patients with SLE. Exclusion criteria included the use of immunosuppressive agents and the presence of health problems and other abnormalities influencing immune responses in healthy volunteers.

The study was approved by the Ethics Committee of Kashan University of Medical Sciences (IR.KAUMS.REC.1394.123) and conducted in accordance with the Declaration of Helsinki. Informed consent was obtained from the participants before entering the study. According to the SD values mentioned in other studies (13, 32), sample sizes were calculated using the following statistical formula:


$$n=\frac{{\left(Z\alpha +Z\beta \right)}^{2}\times \left(S1^{2}+S2^{2}\right)}{{\left(m1-m2\right)}^{2}},$$

α (study accuracy) = 95%,
β (study power) = 80%,
S1 = 1.3,
Zα = 1.96,
Zβ = 0.83.

The mean difference between groups 1 and 2 was (m1 − m2) = 0.95.

### RNA extraction, reverse transcription, and quantitative polymerase chain reaction

For Mina53 expression analysis, total RNAs were isolated from peripheral blood mononuclear cells (PBMCs) using a High Pure RNA Isolation Kit following the manufacturer’s instructions (Roche Applied Science, Penzberg, Germany). RNA yield was determined, and the purity was assessed using a spectrophotometer (NanoDrop 8000 spectrophotometer, Thermo Scientific, Waltham, MA, USA). Complementary deoxyribonucleic acid (cDNA) synthesis was performed using a Transcriptor First Strand cDNA Synthesis Kit according to the manufacturer’s protocol (Roche Applied Science). TaqMan-based real-time PCR assay was carried out using an ABI7700 machine (Applied Biosystems, Foster City, CA, USA) and the TaqMan Universal PCR Master Mix (PerkinElmer, Waltham, MA, USA) according to the manufacturer’s instructions. Each reaction was initiated at 95°C for 30 seconds, followed by 40 cycles of 95°C for 5 seconds and 60°C for 30 seconds. All analyses were performed in duplicate.

Threshold cycle (Ct) and melting curve were automatically generated by the Applied Biosystems software. The expression level of glyceraldehyde-3-phosphate dehydrogenase (GAPDH) was used as an endogenous control to normalize the expression level of each sample. The cycling parameters for GAPDH were the same as those used for Mina53. The comparative ΔCt method was used to measure the quantitative expression level of the Mina53 gene. Primer sequences are shown in **Table 1**.

**Table 1:** Primer sequences for amplification of Mina53 and GAPDH mRNAs in TaqMan-based real-time PCR.

Genes	Forward primer (5′–3′)	Reverse primer (5′–3′)
**Mina53**	GGG ACA CAA CAT TGG GTA TCA TCA	AAC ATG GGC AAT TCA GGC AGA
**GAPDH**	GTG AAG GTC GGA GTC AAC G	TGA GGT CAA TGA AGG GGT C

Mina53, Myc-induced nuclear antigen 53; GAPDH, glyceraldehyde-3-phosphate dehydrogenase.

### The assessments of Mina53 and other laboratory parameters

The serum concentrations of Mina53, complement C3 and C4, anti-double stranded DNA (anti-dsDNA) antibody, ANA, and total hemolytic complement activity (CH50) were assessed in the whole blood (5 mL) from the participants using enzyme-linked immunosorbent assay (ELISA) kits (MyBioSource, San Diego, CA, USA) according to the manufacturer’s instructions. Briefly, 50 µL of the serum was added to the wells of a micro-ELISA plate, and the samples were incubated at 37°C. After a 30-minute incubation, the liquid was removed from each well. The wells were washed five times with a washing solution. To each well, 100 µL of biotinylated detection antibody was added, followed by incubation for another 30 minutes at 37°C. The wells were washed several times with washing solution. Afterward, 100 µL of horseradish peroxidase conjugate working solution was added. The samples were incubated at 37°C for 30 minutes. Substrate solution (100 µL) was added, and samples were kept for 15–20 minutes at room temperature in the dark. Then, the stopper solution (50 µL) was added to each well, and absorbance was read at 450 nm using a spectrophotometer (Vira Teb Tajhiz, Tehran, Iran).

### Statistical analysis

Data analysis was carried out using the SPSS program (v. 20; SPSS, Chicago, IL, USA). The results are represented as mean ± standard deviation (SD). The D’Agostino–Pearson test was used to evaluate the normal distribution of the data. According to the non-normal distribution of the data, the groups were compared using the Mann–Whitney U and Kruskal–Wallis tests. Spearman’s test was used to determine correlation coefficients of the data with non-normal distribution. Multiple binary logistic regression models were used for multivariate analysis. The correlations were evaluated using Fisher’s exact and chi-square tests. Using the receiver operating characteristic (ROC) area under the curve (AUC), an attempt was made to determine the specificity, sensitivity, positive predictive value (PPV), and negative predictive value (NPV) of Mina53 in the diagnosis of SLE and its severity. A p-value less than 0.05 was considered the minimum level of statistical significance.

## Results

Sixty SLE subjects and 30 healthy subjects were enrolled in the study (**Table 2**). There was no significant difference in duration of disease and history of previous treatment between patients with mild and severe SLE (**Table 2**). The clinical, laboratory, and demographic characteristics of all participants are summarized in **Table 2**.

**Table 2:** Demographic, clinical, and laboratory characteristics of all participants of the study.

		Healthy control	Mild SLE	Severe SLE	p-Value
**Age (years)**		35.97 ± 9.9	35.87 ± 9.88	34.5 ± 10.37	0.812
**Duration of disease (years)**		–	4.2 ± 1.51	4.26 ± 1.57	0.868
**Gender**	**Male (%)**	14 (46.7%)	10 (33.3%)	8 (26.7%)	0.257
	**Female (%)**	16 (53.3%)	20 (66.7%)	22 (73.3%)	
**Previous treatment**		–	22 (73.3%)	24 (80%)	0.542
**Response to treatment**		–	Partial response: 4 (13.2%) Good response: 26 (86.8%)	Partial response: 18 (60.4%) Good response: 12 (39.6%)	<0.001
**Disease activity**		–	7.36 ± 0.88	17.7 ± 2.35	<0.0001
**Clinical characteristics**		–	Lupus arthritis: 30 (100%) Malar rash: 6 (19.8%) Thrombocytopenia: 3 (9.9%) Raynaud’s phenomenon: 1 (3.3%) Mouth ulcers: 12 (39.6%) Photosensitivity: 18 (60.4%) Leukopenia: 4 (13.2%) Renal involvement: 3 (9.9%) Abortion: 1 (3.3%) Hemolytic anemia: 1 (3.3%) Lymphadenopathy: 1 (3.3%) Skin lesions: 1 (3.3%) Pleurisy: 1 (3.3%)	Lupus arthritis: 30 (100%) Malar rash: 12 (39.6%) Thrombocytopenia: 6 (19.8%) Pericarditis: 1 (3.3%) Vasculitis: 3 (9.9%) Raynaud’s phenomenon: 2 (6.6%) Mouth ulcers: 15 (50%) Photosensitivity: 13 Leukopenia: 6 (19.8%) Renal involvement: 4 (13.2%) Autoimmune hepatitis: 1 (3.3%) Abortion: 6 (19.8%) Brain stroke: 3 (9.9%) Hemolytic anemia: 4 (13.2%) Lymphadenopathy: 1 (3.3%) Hair loss: 2 (6.6%) Skin lesions: 3 (9.9%)	–
**Mina53 serum levels**		89.3 ± 35.5	174.1 ± 51.9	216.4 ± 48.4	<0.001
**Mina53 gene expression**		6.9 ± 2.04	9.63 ± 1.73	10.77 ± 2.01	<0.001
**Anti-dsDNA (IU/mL)**		15 ± 13	24.63 ± 26.65	190.5 ± 28.75	< 0.0001
**Complement C3 (mg/dL)**		90 ± 16	108.2 ± 24.66	85.53 ± 33.23	< 0.01
**Complement C4 (mg/dL)**		29 ± 23	18.67 ± 8.92	12.65 ± 8.13	< 0.01
**CH50**		130 ± 25	96.27 ± 24.66	101.4 ± 113	0.1

SLE, systemic lupus erythematosus; Mina53, Myc-induced nuclear antigen 53.

### The expression and serum levels of Mina53 in patients with SLE

**Figure 1:**
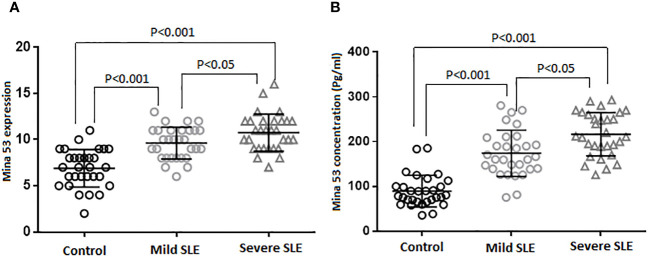
Gene expression and serum concentration of Mina53 protein. **(A)** The expression level of Mina53 in PBMCs from patients with mild SLE (n = 30) and severe SLE (n = 30) and healthy subjects (n = 30) was measured using TaqMan-based real-time PCR. **(B)** The serum level of Mina53 in patients with mild SLE (n = 30) and severe SLE (n = 30) and healthy subjects (n = 30) was measured using ELISA. Data are shown as mean ± SD. ^*^p< 0.05, ^***^p< 0.001. Mina53, Myc-induced nuclear antigen 53; PBMCs, peripheral blood mononuclear cells; SLE, systemic lupus erythematosus.

The results of the current study revealed that SLE patients had a significant increase in Mina53 serum and gene expression levels compared to healthy subjects (p< 0.001, **Table 2**, **Figures 1A, B**). Patients with severe SLE showed higher serum and expression levels of Mina53 than patients with mild SLE (p< 0.05, **Figures 1A, B**).

### The association of Mina53 protein with SLE severity

**Figure 2:**
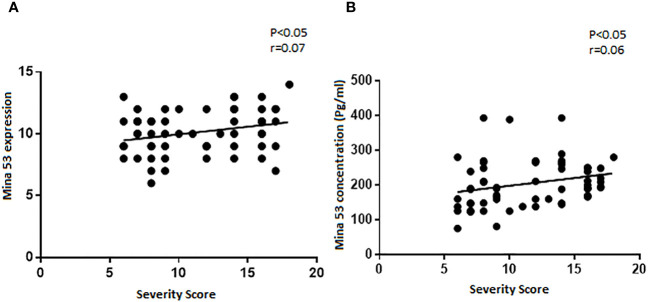
The relationship of SLE severity with gene expression and serum concentration of Mina53. **(A, B)** Statistical tests revealed that the expression and serum levels of Mina53 were significantly correlated to SLE severity. SLE, systemic lupus erythematosus; Mina53, Myc-induced nuclear antigen 53.

Although there were weak correlations between the expression and serum levels of Mina53 and severity of SLE (r = 0.07 and r = 0.06), these relationships were statistically significant (p< 0.05, **Figures 2A, B**).

The logistic regression models showed that the serum and expression levels of Mina53, unlike age and sex, affected the occurrence of SLE (p< 0.01, Model. 1, **Table 3**). Furthermore, it was shown that Mina53 serum and gene expression levels, unlike age and sex, are effective factors in SLE severity (p< 0.001, Model 2, **Table 3**).

**Table 3:** Predictor variables of SLE disease and its severity in multiple binary logistic regression models.

Models	Outcomes	Variables	B^1^	SE^2^	Sig.^3^	Exp (B)^4^
**1**	**SLE disease**	**Mina53 gene expression**	1.488	0.527	0.005	4.426
		**Mina53 serum level**	0.057	0.016	<0.001	1.059
		**Sex**	0.102	1.01	0.92	1.107
		**Age**	−0.089	0.069	0.197	0.915
		**Constant**	−16.73	5.39	0.002	0.000
**2**	**Severe SLE**	**Mina53 gene expression**	0.496	0.178	0.005	1.643
		**Mina53 serum level**	0.024	0.006	<0.001	1.024
		**Sex**	0.456	0.698	0.513	1.578
		**Age**	−0.011	0.029	0.709	0.989
		**Constant**	−9.43	2.68	<0.001	0.000

SLE, systemic lupus erythematosus; Mina53, Myc-induced nuclear antigen 53.

^1^ Coefficient model.

^2^ Standard error of B.

^3^ Significant level (p-value).

^4^ Adjusted odds ratio.

### The relationships of Mina53 serum and expression gene levels with laboratory parameters and demographic information

**Figure 3:**
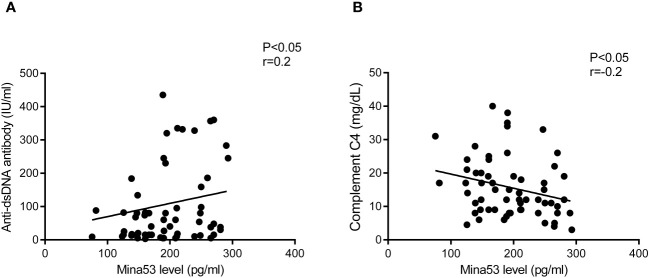
The association of Mina53 value with laboratory factors. **(A)** Mina53 serum level was positively correlated to anti-dsDNA antibody. **(B)** There was a significant negative association between Mina53 value and complement C4 concentration. Mina53, Myc-induced nuclear antigen 53.

Spearman’s test revealed that Mina53 serum level was positively correlated to anti-dsDNA antibody (p< 0.05, r = 0.2, **Figure 3A**). However, there was a negative association between Mina53 value and complement C4 concentration (p< 0.05, r = −0.2, **Figure 3B**). No significant correlation was observed between Mina53 protein level and other laboratory parameters used for SLE diagnosis, such as complement C3, ANA, and CH50 values. Furthermore, Mina53 serum and expression gene levels were not associated with the age and sex of patients with mild and severe SLE.

### Predicting role of Mina53 in determining SLE development and its severity

**Figure 4:**
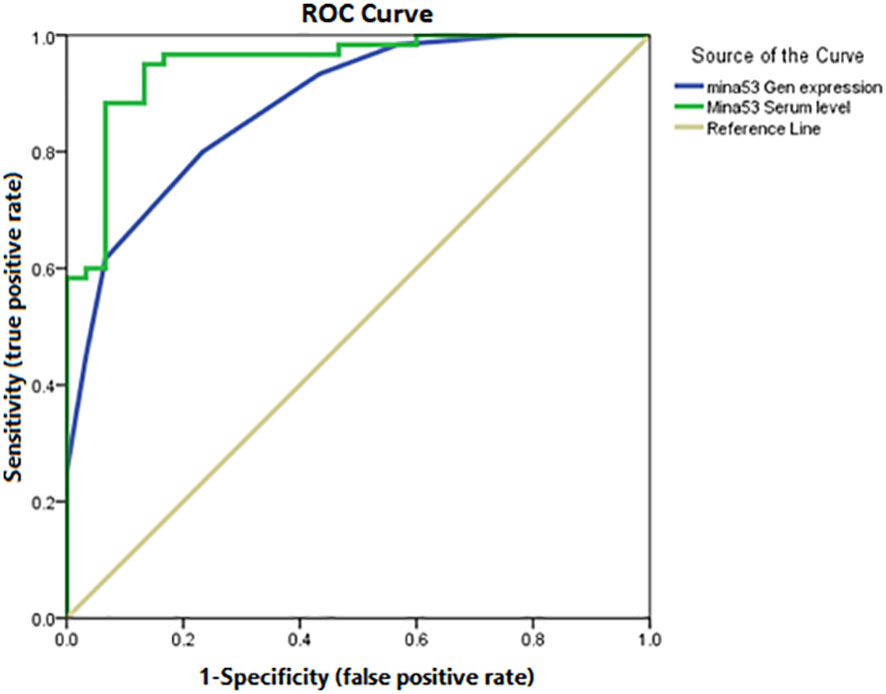
Sensitivity and specificity of serum and expression levels of Mina53 in SLE diagnosis. Mina53, Myc-induced nuclear antigen 53; SLE, systemic lupus erythematosus.

**Figure 5:**
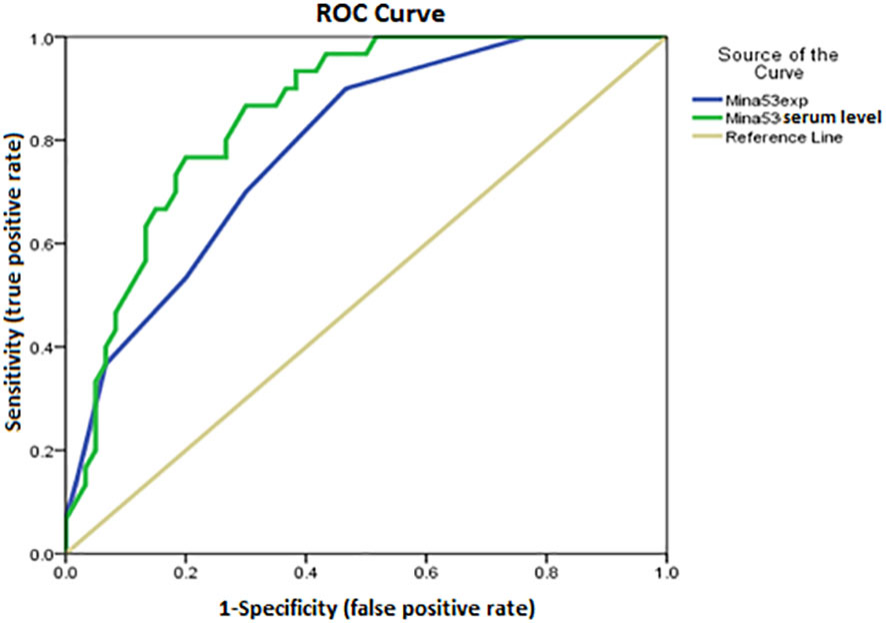
Sensitivity and specificity of serum and expression levels of Mina53 in determining disease severity. Mina53, Myc-induced nuclear antigen 53.

Using ROC AUC, an attempt was made to determine the sensitivity and specificity of Mina53 serum level and its gene expression as diagnostic markers for SLE and its severity. As diagnostic markers for SLE, the highest sensitivity (95%) and maximum specificity (83.3%) were found in the cut-off point of Mina53 serum level equal to 125.4 (AUC = 0.951), while the highest sensitivity (80%) and maximum specificity (76.7%) were observed in the cut-off point of Mina53 expression level equal to 8.5 (AUC = 0.88, **Table 4**, **Figure 4**). As diagnostic markers for severe form of SLE, the highest sensitivity (93.3%) and maximum specificity (60%) were found in the cut-off point of Mina53 serum level equal to 139.5 (AUC = 0.854, **Table 4**, **Figure 5**). The highest sensitivity (90%) and maximum specificity (53.5%) were found in the cut-off point of Mina53 expression level equal to 8.5 (AUC = 0.788, **Table 4**, **Figure 5**). In contrast, the highest sensitivity (100%) and maximum specificity (80%) were observed in the cut-off point of anti-dsDNA antibody level equal to 28.5 (AUC = 0.940, **Table 4**, **Figure 5**).

**Table 4:** Sensitivity, specificity, and predicting values of Mina53 and anti-dsDNA antibody in SLE diagnosis.

Outcomes	Parameters	Cut-off point	AUC	Sensitivity	Specificity	PPV	NPV
**Mild and severe SLE**	**Mina53 serum level**	125.4	0.951	0.95	83.3	91.9	89.3
	**Mina53 gene expression**	8.5	0.88	0.8	76.7	87.3	65.7
**Severe SLE**	**Mina53 serum level**	139.5	0.854	93.3	60	53.8	94.7
	**Mina53 gene expression**	8.5	0.788	0.9	53.3	49.1	91.4
	**Anti-dsDNA antibody**	28.5	0.940	100	80	83.3	100

Mina53, Myc-induced nuclear antigen 53; SLE, systemic lupus erythematosus; AUC, area under the curve; PPV, positive predictive value; NPV, negative predictive value.

## Discussion

Mina53 has a fundamental role in altering the balance between Th17 cells and Tregs, which can participate in the pathogenesis of inflammatory diseases (30). Having considered that immune imbalance is largely related to the development of SLE (3), the expression and serum levels of Mina53 as an important regulator of inflammation were assessed in SLE patients with various levels of severity.

The results of this study revealed that patients with SLE had a significant increase in the serum level of Mina53 compared to healthy subjects, which was accompanied by the enhanced gene expression of this protein. After adjusting the effects of confounding factors such as age and sex, our data indicated that the expression and serum levels of Mina53 were significantly higher in patients with severe SLE than in patients with mild SLE. In our knowledge, these findings, for the first time, provide evidence to indicate that Mina53 serum and gene expression levels were significantly associated with SLE severity. In line with the possible roles of Mina53 in the development and severity of disease, previous studies have reported that SLE patients possess an increased frequency in Th17 cells, which is significantly associated with disease activity and severity (9, 10). Other studies have shown that an immune imbalance in SLE subjects participates in symptom exacerbation through shifting immune response from Tregs to Th17 cells (29, 30, 33). In this regard, SLE patients showed a dual role for Mina53 in increasing the expression of cytokines and transcription factors of Th17 cells and reducing the expression of Foxp3 as a main transcription factor of Tregs (30). Others have indicated that Mina53 induces the infiltrations and functions of Th17 cells and macrophages and inhibits the suppressive effects of Tregs (30, 34). Animal studies have shown that genetic deficiency of Mina53 impairs the expression of cytokines and transcription factors of Th17 cells and enhances the expression of Foxp3 of Tregs. Similar studies on diseases with immunopathology have demonstrated that ablation of Mina53 provides a protective impact on silica-induced lung fibrosis, which is associated with impaired Th17 and elevated Treg infiltration in the lung (30). Moreover, several studies have shown that genetic changes related to over-expression of Mina53 may enhance the risk of the development of asthma (35). In addition to the roles of Mina53 in the development of autoimmunity, there are several studies pointing to its impacts on oncogenesis (36). Mina53 has a well-known role in lung cancer, which may correlate to its effects on the chronic inflammatory response, cell differentiation, and DNA repair (36). Furthermore, it is shown that the increased expression of Mina53 may play a fundamental role in the development of human pancreatic cancer (32).

A series of papers have confirmed that Mina53 is involved in DNA replication and DNA damage response. Mina53 participates in regulating DNA replication and its stability through physical interaction with several DNA replication proteins (37). Therefore, aberrant DNA repair could be an important underlying mechanism of autoimmunity such as SLE (38). Along with these observations, our findings propose that Mina53 may be a main regulator in the occurrence of SLE and its severity.

In the next step, the correlation of Mina53 with SLE development, disease severity, demographic characteristics, and laboratory parameters used for disease detection were evaluated. Statistical analyses revealed that the expression and serum levels of Mina53 had significant correlations with the severity of SLE, although these relationships were statistically weak (r = 0.07 and r = 0.06) due perhaps to low sample size. Additionally, the logistic regression models showed that the serum and gene expression levels of Mina53, unlike age and sex, are effective factors in the occurrence and severity of SLE. Other results of the current study indicated that the serum concentration and gene expression of Mina53 were not influenced by the age and gender of patients with mild and severe SLE, which is consistent with the results of a study conducted on Mina53 expression in patients with pancreatic cancer (32). Tan et al. reported that the expression of Mina53 was not associated with demographic characteristics, such as sex and age, of patients suffering from pancreatic cancer (32). In an attempt to determine the relationship of Mina53 with laboratory parameters, we observed that Mina53 protein level was positively correlated to anti-dsDNA antibody. In contrast, a significant negative association was observed between the Mina53 value and complement C4 concentration. Having considered that the increased level of anti-dsDNA antibody and reduced value of complement C4 are largely related to the pathogenesis and severity of SLE (39), our findings provide further confirmation regarding the association between Mina53 and the development and severity of SLE.

To confirm the predicting roles of expression and serum levels of Mina53 in the development and severity of SLE, ROC AUC was used to determine the specificity and sensitivity of Mina53 in SLE diagnosis and its severity. The results indicated that the cut-off point of Mina53 serum level equal to 125.4 (AUC = 0.951) and the cut-off point of Mina53 expression level equal to 8.5 (AUC = 0.88) had the highest specificity and maximum sensitivity for SLE diagnosis. However, the cut-off point of Mina53 serum level was equal to 139.5 (AUC = 0.854) and the cut-off point of Mina53 expression level was equal to 8.5, showing the highest specificity and maximum for determining SLE severity. Although Mina53 serum and gene expression levels have lower diagnostic efficiency than anti-dsDNA antibody value (AUC = 0940 versus AUC = 0.854 and 0.788) in SLE severity, the findings of the current study suggest that Mina53 serum level, unlike its expression level, has higher efficiency for diagnosing SLE disease than anti-dsDNA antibody value (AUC = 0951 versus AUC = 0940). Nonetheless, the current study has some limitations including I) the lack of suitable functional assays, which provide supplementary information on the immunologic mechanisms of Mina53 in SLE, and II) the absence of experiments studying epigenetic and environmental factors that make some changes in the expression and production of Mina53 protein. Therefore, it is recommended that further studies be carried out to clarify the possible effects of environmental and epigenetic variables on Mina53.

## Conclusion

The results of the present study for the first time provide evidence to indicate that the changes of serum and expression levels of Mina53 may significantly affect the development of SLE and its severity, which are independent of sex and age. These values may be used as a diagnostic marker for SLE development and its severity. However, it should be noted that further studies with larger sample sizes are required to confirm the results of this study.

## Data availability statement

The original contributions presented in the study are included in the article/supplementary material. Further inquiries can be directed to the corresponding authors.

## Ethics statement

The studies involving humans were approved by The Ethics Committee of Kashan University of Medical Sciences (IR.KAUMS.REC.1394.123). The studies were conducted in accordance with the local legislation and institutional requirements. Written informed consent for participation was not required from the participants or the participants’ legal guardians/next of kin because Informed consent was taken before taking part in the study.

## Author contributions

BZ: Funding acquisition, Writing – original draft. ED: Investigation, Writing – original draft. HA: Formal Analysis, Validation, Writing – original draft. HN: Methodology, Project administration, Supervision, Writing – review & editing. HM: Methodology, Writing – review & editing.
